# Lateralisation of the white matter microstructure associated with the hemispheric spatial attention dominance

**DOI:** 10.1371/journal.pone.0216032

**Published:** 2019-04-26

**Authors:** Krisztián Kocsis, Gergő Csete, Zsombor Erdei, András Király, Nikoletta Szabó, László Vécsei, Zsigmond Tamás Kincses

**Affiliations:** 1 Department of Neurology, Albert Szent-Györgyi Clinical Center, University of Szeged, Szeged, Hungary; 2 Neuroscience Research Group of the Hungarian Academy of Sciences and University of Szeged, Szeged, Hungary; 3 Department of Radiology, Albert Szent-Györgyi Clinical Center, University of Szeged, Szeged, Hungary; McLean Hospital, UNITED STATES

## Abstract

**Objectives:**

Healthy people have a slight leftward bias of spatial attention as measured on the Landmark task. Former studies indicated that lateralisation of brain activation contributes to this attentional bias. In this study we hypothesised that if the spatial bias was consistent over several measurements there would be structural background of it.

**Methods:**

Reproducibility of the spatial bias of visuo-spatial attention was measured in twenty healthy subject in a Landmark task over three consecutive days. In order to evaluate the correlation between the spatial attentional bias and the white matter microstructure high angular resolution diffusion MRI was acquired for each subjects. The Track Based Spatial Statistics method was used to measure the hemispheric differences of the white matter microstructure. Probabilistic tractography was used to reveal the connection of the identified regions.

**Results:**

The analysis showed correlation between the behavioural scores and the lateralisation of the white matter microstructure in the parietal white matter (p<0.05, corrected for multiple correlations). Higher FA values on the left are associated to rightward bias. The parietal cluster showed connectivity along the superior longitudinal fascicle on one end to posterior parietal cortex and anteriorly to the putative frontal eye field. From the frontal eye field some of the fibres run towards the nodes of the dorsal attention network to the intraparietal suclus, while some of the fibres travelled toward to ventral attention network to the temporo-parietal junction.

**Conclusions:**

These results indicate that the structural integrity dorsal fronto-parietal network and the connection between the dorsal and ventral attention networks are responsible for the attentional bias in normal healthy controls.

## Introduction

Lateralised brain functions are thought to increase the brain capacity by processing the information parallel and complementary [1, 2]. Since the early work of Broca and Wernicke showing left sided brain damage being associated with problems of language production and comprehension, the functional and structural asymmetries of the brain are in the centre of research [3, 4]. Lesion-symptom mapping studies found similar lateralisation for example for visuo-spatial attention [5] and emotional expression [6]. With the rapid development of non-invasive brain imaging approaches our understanding of the hemispheric lateralisation increased enormously. Series of studies described the detailed functional anatomy of the language system [7], and lateralised motor functions [8]. It was also hypothesised that functional lateralisation is related to structural hemispheric differences. Consequently, structural imaging investigations shed light on the innate or acquired lateralised grey matter structure behind handedness [9, 10] and language [11–14]. While lateralised grey matter structure is unquestionable, it cannot fully explain the functions [14, 15]. Variation of the micro and macrostructural measures of the white matter connecting the functional network nodes might account for at least some of the functional asymmetries [16]. Hence, lateralised white matter organisation was shown in the language system as well as in the motor system [11, 17–19].

One of the interesting lateralisation in the brain is related to the visuo-spatial attention. It is well known in clinical practice that right-sided brain damage frequently causes spatial neglect syndrome [5]. Patients with hemineglect have a reduced capacity to pay attention to the left visual field and neglect the left side of the space. Voxel-based lesion symptom mapping studies attempting to find the region responsible for hemispatial neglect concluded that right perisilvian cortical regions such as the right temporo-parietal junction, superior temporal cortex, inferior parietal lobule and insula are the most common substrate of neglect [20–23]. Furthermore, neglect may develop because of the damage of the inferior and middle frontal gyrus, the ventral premotor cortex on the right [24]. Not surprisingly, further studies indicated that the damage of the right perisilvian white matter, the superior longitudinal fasciculus and the inferior occipito-frontal fasciculus connecting the above mentioned frontal and parietal regions are key factors in developing neglect [25]. Creating a virtual lesion in the right posterior parietal cortex by transcranial magnetic stimulation Fierro and colleagues not only reproduced the attentional deficit but demonstrated that it is crucial in the processing of spatial information 150ms after the stimulus presentation.

The lateralised spatial-attention system is also supported by the bias seen in linebisection or in Landmark tasks in healthy subjects, known as pseudoneglect. In the former, the subject is asked to bisect a horizontal line in the middle. Neurologically intact subjects tend to err slightly to the left [26]. In the latter, perceptual version, the longer segment have to be identified of a horizontal line bisected close to the middle and normal healthy tend to overestimate the size of the leftward segment [27, 28].

To account for these observations two alternative hypotheses are competing in the literature: (i) According to the hemispatial theory right hemisphere directs attention to both visual hemifields, whereas left hemisphere deals with the right hemifield only [29–31]. This constellation could explain the more frequent occurrence of neglect syndrome with right hemisphere damage. (ii) The interhemispheric competition theory states that each hemisphere directs attention to the contralateral visual field that is under reciprocal inhibition and the strength of this attentional bias is stronger in the left hemisphere [32].

Functional imaging studies of spatial attention revealed two interacting networks [33, 34]: (i) the largely bilateral dorsal fronto-parietal network including the frontal-eye-field and the intraparietal sulcus and the (ii) right lateralised ventral attentional system including the temporoparietal junction and the ventral frontal cortex. As regarding the function of these two systems the bilateral dorsal is responsible for goal directed, endogenous, top-down modulation of lower level centres, while the ventral right lateralised system is detecting salient stimuli and stimulus driven shifts of attention.

Functional magnetic resonance imaging and event related potential studies showed that lateralised activation can be found in a Landmark task in a similar right lateralised fronto-parietal network [34–38]. Szczepanski and her colleagues showed that regions within this fronto-parietal network generate spatial bias and the sum of that attentional weights constitute the overall bias [39]. In an fMRI task the sum of these attentional weights contributed to the individual’s bias on the line bisection task [40]. This later, and also some former studies call attention to the interesting fact that while most of the normal healthy subject has a leftward bias on spatial attention tasks, a minority of people have no bias or even to the opposite direction.

While there is detailed information about the brain function behind the pseudoneglect phenomenon, the information on the structural background is sparse. Since the attentional bias of an individual seems to be highly consistent over time [41], it is indeed likely that there is a hemispheric lateralisation of the brain structure behind this feature. It is known that function is deeply rooted in the anatomical structure, and behavioural performance is strongly influenced by the properties of the underlying brain structure. It was shown that correlation between the individual structural variability and behavioural performance can identify the involved neuroanatomical structures [42]. The microstructural integrity of the white matter as defined by diffusion tensor imaging is capable of revealing the coupling of structure and function [43–45]. Accordingly, Thiebaut de Schotten showed that the relative size of the right superior longitudinal fasciculus is correlated with the behavioural bias on the line-bisection task [46], which results were later replicated by Cazzoli and colleagues [47].

While the merit of the above mentioned studies is unquestionable there are factors that needs further investigation. These two studies investigated the motor version of the line bisection task. It was formerly showed that the motor and perceptual versions (line bisection vs. Landmark) generally yield different results and activates different networks [35, 48–50]. Hence in the current experiment we used the perceptual version of the task.

Furthermore, the above mentioned two studies restricted their analysis to prespecified white matter tracts and correlated the tract volume and average diffusion property (hindrance modulated orientation anisotrophy) with the attentional bias. While, based on the results of former investigations it is certainly reasonable to investigate the various branches of the superior longitudinal fascicle, a whole brain analysis could further strengthen the results. Hence, in the current study we considered all the white matter fibres, but to avoid the registration bias we restricted the analysis to the alignment-invariant white matter skeleton representing the middle of the fibre tracts [51].

In this study we set out to find lateralisation of the integrity of the white matter fibres that underlines the pseudoneglect phenomenon in a perceptual Landmark task. As a first step we investigated the reproducibility of the pseudoneglect in a Landmark task, to see if it is a suitable measure to identify the structural background of the phenomenon. In a second diffusion tensor MRI study we correlated the lateralisation of the white matter integrity and the direction of the spatial bias in a Landmark task.

We hypothesised that we would find asymmetric organisation of the white matter tracts that correlated with the behavioural bias on the Landmark task.

## Methods

### Subjects

Twenty healthy subject participated in the studies (mean age = 25.85±2.94 years). All subjects were right handed according to the Edinburgh handedness inventory (mean score = 9.21±2.08). None of them suffered from any neurological or psychiatric diseases. All of them had normal or corrected-to-normal (20/20) visual acuity and good colour vision. The study was approved by the ethical committee of the University of Szeged (Ref. no.: 87/2009) and all study participants gave their written informed consent in accordance with the Declaration of Helsinki.

### Stimulus presentation and experimental paradigm

**Fig 1:**
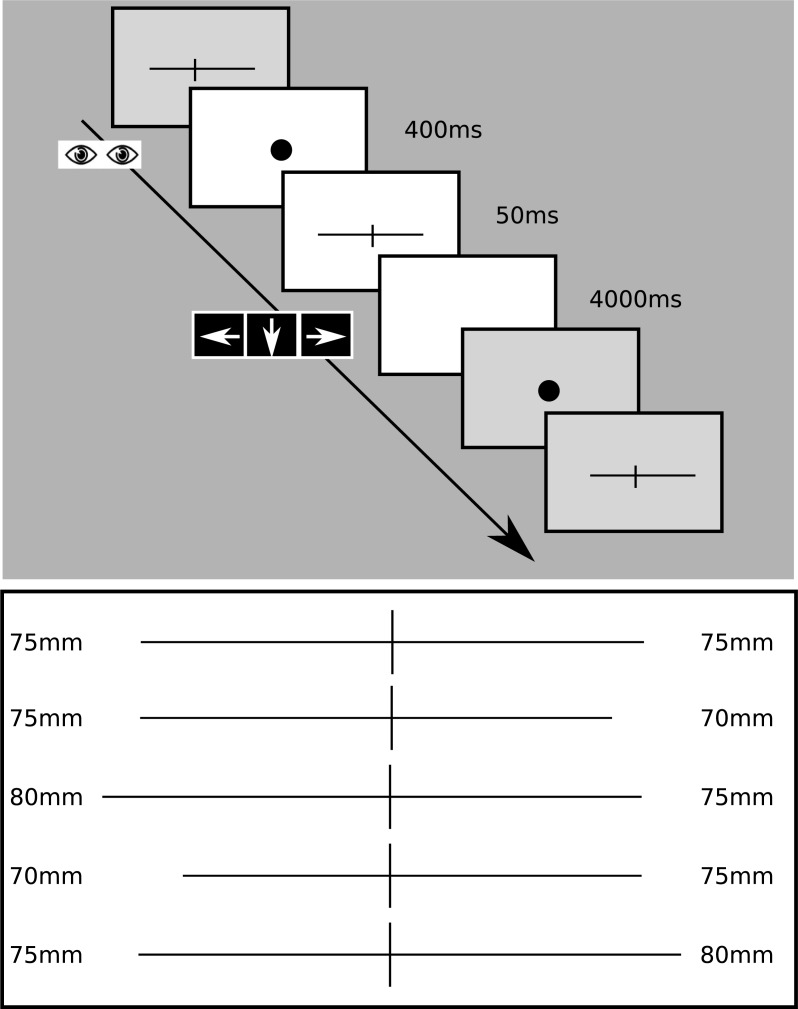
Landmark task. Fixation dot appeared for 400ms, during which fixation was monitored. The stimuli (bisected line) were presented for 50ms. The interstimulus interval was 4000ms, during which the subject had to indicate which segment of the bisected line was longer by pressing left or right arrow keys or the down key for equal segments. Line1: mid-bisected (each segments 75 mm), Line2: right bisected (left segment: 75mm, right segment: 70mm), Line3: extreme right bisected (left segment: 80mm, right segment: 75mm), Line4: left bisected (left segment: 70mm, right segment: 75mm), Line5: extreme left bisected (left segment: 75mm, right segment: 80mm).

The Landmark task was presented on a Tobii Pro TX300 23” Eye Tracker TFT monitor with 1920x1080 maximal screen resolution to achieve accurate fixation during the task. The custom-made Landmark-task was programmed in Matlab R2012b, with Psychophysics Toolbox Version 3.0.10 (PTB-3) and consisted of 1mm thick black horizontal lines presented on the middle of the screen. The horizontal lines were transsected with a 10 mm high black vertical bar. The vertical line was set at the centre of the screen. In the task five different lines were used with different lengths (Fig 1): (i) bisected exactly in the middle, (ii-iii) left elongated, (iv-v) right elongated, iii. and v. being more extreme.

The participants were seated in a chair in front of the monitor. The distance of the participant’s eyes from the screen was 55–60 cm. The correct fixation during the task was controlled with the Tobii Pro TX300 Eye Tracking system (gaze sampling rate: 300 Hz; operating distance: 50–80 cm; binocular and dark pupil tracking technique). Before stimulus presentation the participants were asked to fixate a central fixation dot in the middle of the screen. The fixation was checked for 400 ms. In case of correct fixation the fixation dot disappeared and the stimuli were presented for 50 ms duration. The stimuli were followed by a white blank screen for 4000 ms intertrial interval after which the next fixation dot appeared. After the stimulus presentation the participants were asked to make forced-choice decision about the respective length of the two segments of the bisected lines with three keyboard button response possibilities: *left*–left longer, *down–*equal lengths and *right* arrow key–right longer. They were asked to judge which segment of the previously bisected line is longer and press the appropriate button. The performance of the subjects was evaluated according to Fierro as follows: 0 = correct response; 1 and 2 = rightward errors due to left under-evaluation; -1 and -2 = leftward errors due to right under-evaluation [28] (Table 1).

**Table 1:** The scoring system of the attentional bias in the Landmark task. The second column depicts the length of the left and right segment of the stimuli. The scores associated with the response in the various line length are shown in column 2–4.

	Length of Left/Right segments	Response		
		Left longer	Equal	Right longer
Line 1	75/75mm	-1	0	1
Line 2	75/70mm	0	1	2
Line 3	80/75mm	0	1	2
Line 4	70/75mm	-2	-1	0
Line 5	75/80mm	-2	-1	0

The five different lines were presented in random order.

In the reproducibility experiment 50 stimuli were presented all five lines repeated 10 times. The measurements were repeated in three consecutive days.

### Statistical analysis of the reproducibility experiment

Statistical analyses were performed using the Statistical Package for Social Sciences (SPSS 20.0.0 for OS X, SPSS Inc., http://www.spss.com). Intraclass correlation coefficient (ρ) analysis was used for the reliability measures:

$$\rho =\frac{{MS}_{BS}-{MS}_{Ws}}{{MS}_{BS}+(k-1){MS}_{WS}}$$

where MS_BS_ is the between subject mean of squares and MS_WS_ is the within subject mean of squares and k is the number of observations. With the interpretation of the reproducibility we followed Cicchetti’s guideline.

### MRI acquisition

Neuroimaging data acquisitions were carried out on a 1.5 T GE Signa Excite HDxt MR Scanner (GE Healthcare, Chalfont St. Giles, UK) within one month of the behavioural testing. Three-dimensional spoiled gradient echo images (FSPGR: echo time [TE]: 4.1 ms; repetition time [TR]: 10.276 ms; matrix: 256x256; field of view [FOV]: 25cmx25cm; flip angle: 15°; in-plane resolution: 1mmx1mm; slice thickness: 1mm) and 60-direction diffusion-weighted images with 6 non-diffusion-weighted reference volumes (TE: 93.6 ms; TR: 16.000 ms; matrix: 96x96; FOV: 23cmx23cm; flip angle: 90°; in-plane resolution: 2.4mmx2.4 mm; slice thickness: 2.4mm; b: 1000 s/mm^2^; number of excitations [NEX]: 2; array spatial sensitivity encoding technique [ASSET]) were acquired.

### Correlation of diffusion parameters with behavioural measures

Diffusion data were corrected for eddy currents and movements artefacts by twelve degree of freedom affine linear registration to the first non-diffusion-weighted reference image. An algorithm included in FMRIB’s Diffusion Toolbox (FDT) of FSL (v.4.0) fit diffusion tensors at each voxel [52]. Fractional anisotropy (FA) was computed for the whole brain. In order to reduce the possible errors arising from misalignment of the images, we used the Tract Based Spatial Statistics (TBSS) method [53]. All subjects' FA images were aligned into a common space, using the non-linear registration tool, FNIRT, which use a b-spline representation of the registration warp field. A mean FA image was created and the threshold set at FA = 0.2, deriving a mean FA skeleton that represented at the centres of all tracts common to the group. In order to test for asymmetries in diffusion characteristics we projected the data to a symmetric skeleton with FSL’s *tbss_sym* algorithm. According to this algorithm the original asymmetric skeleton is thickened than the mean FA image is flipped along the y axis and flipped and non-flipped images are averaged. This averaged, symmetrised mean FA image is fed into the skeletonisation program and then masked by the dilated original skeleton. Finally, this skeleton is flipped along the y axis also and masked by the original non-symmetrised skeleton. The prelaligned FA data are projected onto the symmetrised skeleton, left-right flipped and the resulting images are subtracted from the non-flipped. Since the same information present on the two sides of the images the right side is masked out and only the left side is subjected to further voxel-wise cross-subject statistics and presented in the results. Modelling and inference using standard general linear model (GLM) design set-up was accomplished using permutation-based cluster analysis (n = 5000) as implemented in FSL software package [54]. The design encoded for the average spatial bias scores across the three measurements on consecutive days. Age and gender was used as nuisance variables. Statistical thresholding was carried out with Threshold Free Cluster Enhancing and all results were corrected for multiple comparisons (family-wise error).

### Structural connectivity

Connectivity of the regions showing significant correlation with attentional bias was defined by probabilistic tractography (FDT, part of FSL: www.fmrib.ox.ac.uk/fsl/fdt). A Multifibre diffusion model was fitted that estimates probability distribution on the direction of 1 or more fibre populations at each voxel [55]. Probabilistic tractography was then performed from any brain voxel by tracing streamline samples through these probabilistic distributions of fibre direction. For tractography, we generated 5000 streamline samples from each seed voxel to build up a connectivity distribution. The number of these samples passing through each brain voxel is interpreted as proportional to the probability of connection to the seed voxel. By fitting a multifibre model to our diffusion data, we were able to trace pathways through regions of fibre crossing [55]. Seed masks were the binary masks of the suprathreshold clusters of the TBSS analysis. The result of the tractography was standardised by the total number of generated tracts (waytotal), thresholded at 10% probability. Finally to reveal a group level connectivity map the individual standardised, thresholded maps were registered to standard space, binarised and summed over subjects.

## Results

### Reproducibility

In our Landmark task, on average the subjects judged the left segment of the line slightly longer (mean score: -4.48), similarly to the findings of Fierro and co-workers [28].

**Fig 2:**
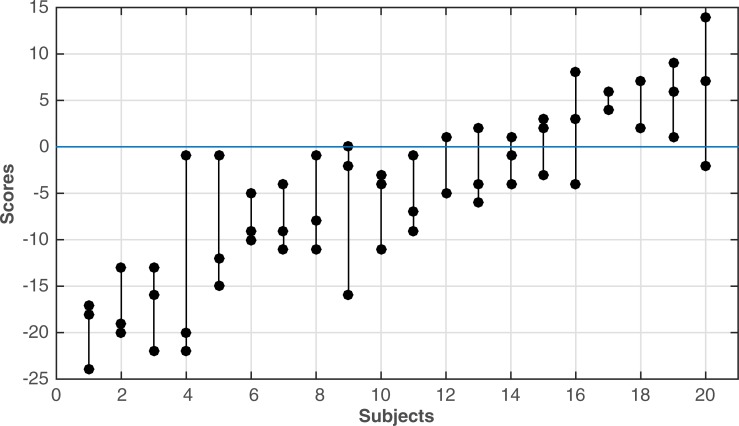
Reproducibility of the spatial bias. The bias scores of the three consecutive measurements plotted for all twenty subjects. The subjects are ordered according to the spatial bias. Where there are only two data points, scores from two measurements were virtually inseparable.

The reproducibility of the attentional bias as measured with Landmark task was evaluated by repeating the task on three consecutive days. The intraclass correlation was ICC = 7.44 (CI: 0.547–0.879), which according to Cicchetti is good-excellent reproducibility (Fig 2).

### Correlation of white matter integrity and attentional bias

In our analysis we tested if the lateralisation of the white matter microstructure correlates with the subjects’ personal spatial attentional bias as measured on the Landmark task in three consecutive days. In order to do so we calculated the hemispheric differences of fractional anisotropy in a symmetric white matter skeleton and correlated the difference with the average spatial bias scores. In our symmetrised-flipped-subtracted images higher values represented higher values on the left in the original space. Positive correlation means higher FA values on the left comes along with more positive spatial bias scores (rightward bias/neglecting the left side of the space). Looking at it from the other direction leftward bias (neglecting the right side of the space) correlate with higher FA values in the right hemisphere (negative values in the subtracted FA image).

**Fig 3:**
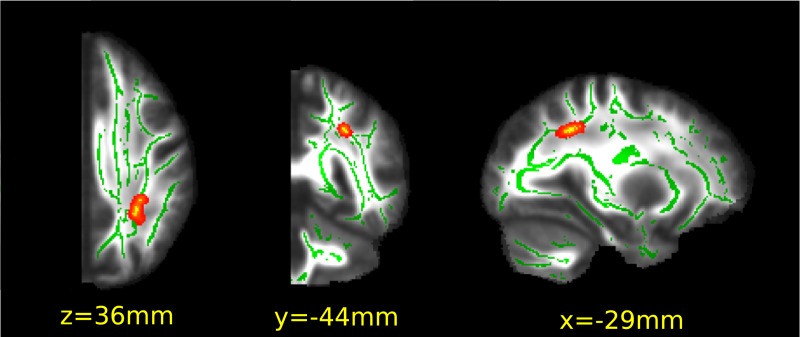
Correlation of the lateralisation of white matter microstructure with the behavioural spatial bias. Statistical Images are overlaid on the FMRIB58_FA standard image. The mean FA skeleton, thresholded at 0.2, is depicted in green shades. Significant cluster is indicated in red-to-yellow (p<0.05, corrected for multiple correlations). A thickened version of the significant cluster is used to facilitate visualization. Positive correlation in the parietal lobe indicates leftward bias correlating with higher FA in the right hemisphere.

Our analysis showed that there was a cluster of positive correlation in the parietal white matter (peak p-value = 0.04, x = -29mm, y = -44mm, z = 36mm, 49 voxels). There was no negative correlation (Fig 3).

### Connectivity of the correlations

**Fig 4:**
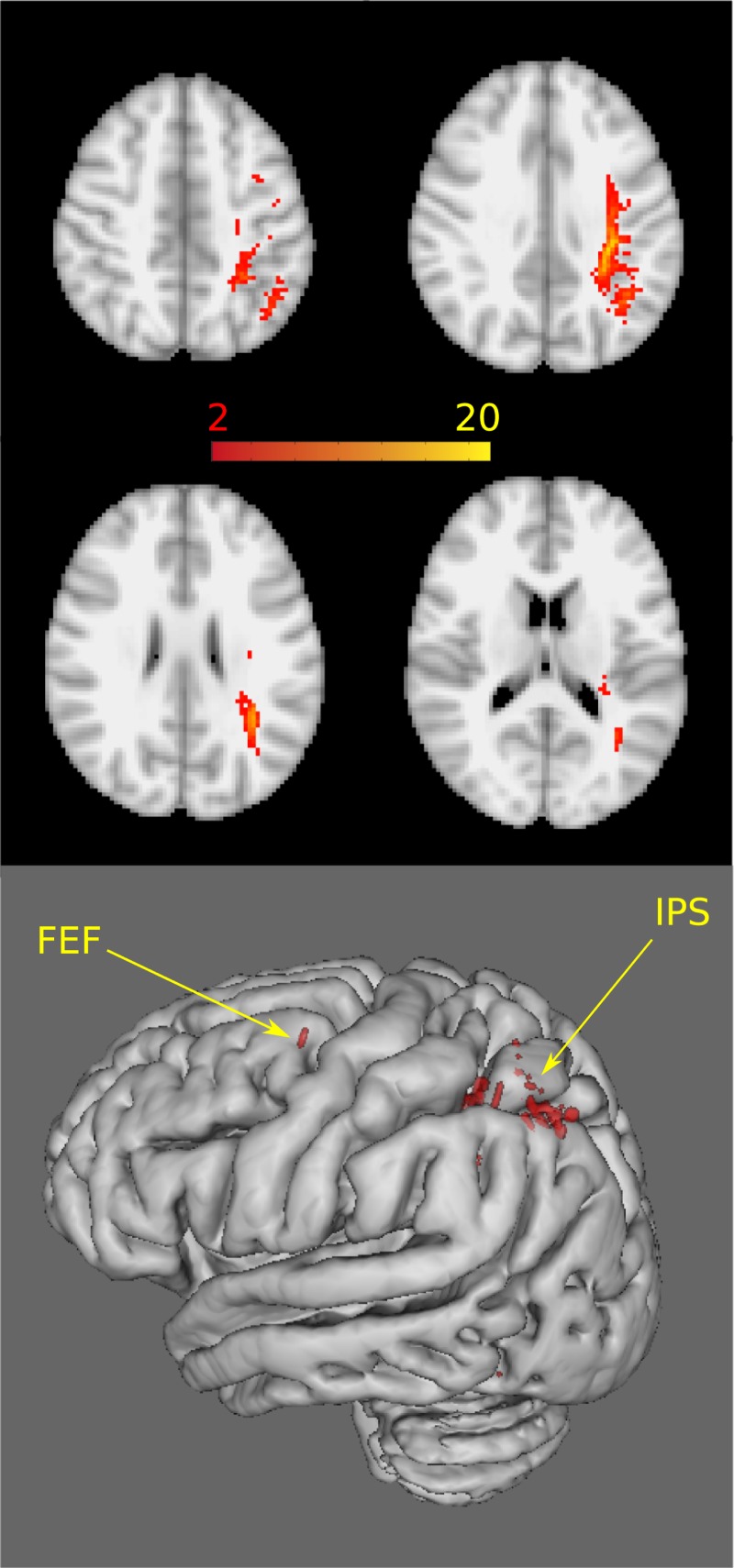
The connectivity of the cluster showing correlation between FA and behavioural data. In the upper section axial slices at standard space coordinates z = 46mm, 36mm, 26mm and 16mm are shown. In the lower section the cortical projections of the tracks are shown. FEF: frontal eye field, IPS: intraparietal sulcus. The binary cluster masks were used as seed mask for each subject. Five thousand streamline samples from each seed voxel were drawn to build up a connectivity distribution that was standardised by the total number of generated tracts, thresholded at 10% and binarized. Population connectivity maps were derived for controls by adding these masks together and thresholding at two (Pathways passing through the given voxel in at least two subjects).

The parietal cluster showed connectivity along the superior longitudinal fascicle on one end to posterior parietal cortex and anteriorly to the putative frontal eye field at the junction of the superior frontal sulcus and the precentral sulcus. In the posterior parietal lobe the tracks run under the bottom of the intraparietal sulcus. Connection travelled to the lateral and medial bank of the intraparietal sulcus and towards the inferior parietal lobule and to the temporoparietal junction (Fig 4).

## Discussion

On average, our subjects showed a leftward attentional bias on the Landmark test. In the reproducibility study we showed that this distribution of spatial attentional bias is consistent over three measurements. This lead us to hypothesis that this spatial attentional bias is hardwired in the brain. Consequently, we found lateralisation of microstructural integrity in the parietal white matter. More organised white matter in the right parietal white matter correlated with a leftward spatial bias (judging the right segment of the line longer). The tractography initiated from this particular seed region identified white matter tracks between the dorsal frontal region and the posterior parietal cortex, regions which are consistently reported as key hubs of attentional network.

Our study has replicated the results of Fierro et al. [28] showing overestimation of the leftward segment of a bisected line on average in a healthy population. Most of the studies reported leftward spatial bias, classically known as pseudoneglect [28, 56–58]. However, there are several studies, which reported a rightward overall bias of their study population [40, 59]. Importantly, the spatial bias is dependent on the task (line bisection vs. Landmark) [35, 48–50] and even the task instruction has a significant effect [60]. Similarly to former studies, our results point out that direction of spatial bias is more of a spectrum. Szczepanski and colleagues showed that the individual variation of attentional bias is in close correlation with the lateralisation of brain activation in the strategic regions of the fronto-parietal network [40]. Importantly, the attentional bias was consistent over several days in our study, which suggests that it is not an immediate result of a constantly changing cerebral activity, but at least partially hardwired in our brain. Indeed, our DTI study proved our hypothesis, higher white matter integrity in the fronto-parietal white matter comes along with attentional bias to the contralateral side. These results speak for the validity of the interhemispheric competition model. Our results indicate that a more integrated structure, which might provide a base for a more coherent functional activation, is overdriving the function of the contralateral hemisphere.

As regarding the spatial location of the correlation we found attentional bias correlating with the white matter integrity in the parietal lobe, in the tracks connecting the posterior parietal lobe and the putative frontal eye field. Former studies of spatial attention revealed two interacting networks: (i) the largely bilateral dorsal fronto-parietal network including the frontal-eye-field and the intraparietal sulcus and the (ii) right lateralised ventral attentional system including the temporoparietal junction and the ventral frontal cortex. As regarding the function of these two systems the dorsal is responsible for goal directed, top-down modulation of lower level centers, while the ventral system is detecting salience stimuli. According to the hypothesis of Corbetta and Shulman there is a delicate interaction between these two systems [34]. In terms of connection between these regions segregated branches of the superior longitudinal fascicle were implicated [46]. The most dorsal branch (SLF I) connects the nodes of the dorsal attention network, while the projections of the most ventral part (SLF III) is a link between the nodes of the ventral attention network. A third branch (SLF II) of the superior longitudinal fascicle partially overlaps with the dorsal and the ventral attention network, connecting the inferior parietal lobule/temporo-parietal junction and the frontal eye-field. This structural link might suggest a communication between the two systems, with redirection of the goal-directed attention mediated by the dorsal network to the events identified as salient by the ventral network [34, 46]. In our investigation the tracks running from our seed connects the frontal eye-field and the intraparietal sulcus, however a minority of fibres travels down towards the temporo-parietal junction. It is also important to note that the spatial coordinates of SLF II as presented by Thiebaut de Schotten coincide with our tracks. These indicate that the dorsal fronto-parietal network (SLF I) and the connection between the dorsal and ventral attention network (SLF II) is the responsible for the attentional bias in normal healthy controls.

It has to be pointed out that the background of pseudoneglect in healthy and the pathological processes leading to neglect not necessarily coincide; variable disturbance of the delicate balance between regions and networks might lead to neglect. Corbetta and Shulman concluded that hemispatial neglect is more likely to be the result of the dysfunction of the ventral attention network [34]. Hattori and colleagues however, showed that various phenotypes of neglect might come around after the damage of the ventral and dorsal attention networks or the connections between these networks [61].

Function and structure are tightly interconnected. A higher FA in the corpus callosum was related to a better bimanual coordination [62]. The integrity of white matter microstructure around Broca’s area was related to the successfulness of artificial grammar learning [63]. Higher microstructural integrity in the fornix was found to be associated with better recollection memory [64]. The rate of visuo-motor adaptation was related to the microstructure of the superior cerebellar peduncle, a structure containing fibres connecting the cerebellum and premotor cortex [65]. We showed that audio-visual integration correlates with the microstructural integrity of fibres in the ‘where’ and ‘what’ visual pathways in low and high contrast conditions respectively [45]. Motion detection threshold was found to be correlated with the diffusion properties of the fibres connecting frontal and parietal association areas, putative attention related regions [44].

The pre-existing structure, which is defining a behaviour [66] and the plasticity altering the brain structure [67–69] is probably jointly responsible for the connection.

The exact histological properties that is leading to the variation in diffusion properties, which has a relevance in certain behavioural performances is not yet clear, but nerve diameter and myelination, which relate to the microstructure measurable by DTI, can relate to the conduction velocity and the dispersion of the neuronal signal [70, 71]. In particular, the precisely paced activity of remote centres in large-scale networks is critical in the appropriate functional performance. One crucial factor of such precise timing might well be the myelination that is a property influencing our diffusion measures [72].

### Limitation

Our study is clearly not without limitations. Direct comparison of the microstructural background of the line bisection and Landmark task could give further insight into the physiology of pseudoneglect. While the effect most probably would not be significant, poststimulus masks could have been used to avoid the aftereffect. The extension of the study to patient populations with subtle white matter damage (eg. multiple sclerosis) could enhance the variability in the data, that could strengthen the analysis. The tight interconnection of structure and function could have been better elaborated by acquiring functional data during the behavioural task.

## Conclusions

Our and former results indicate that two major factors, (i) the concomitant functional activations developing on the skeleton provided by the (ii) brain structure on the macroscopic as well as on the microscopic scale contribute to the behavioural variation. The parallel investigation of this two levels of the function-structural integration is essential to understand the complexity of brain function.
